# A120 METHYLGLYOXAL (MGO) AS A SUBSTRATE FOR LACTYLATION IN ESOPHAGEAL SQUAMOUS CELL CARCINOMA

**DOI:** 10.1093/jcag/gwae059.120

**Published:** 2025-02-10

**Authors:** M Hamilton, J Douchin, M Fréchette, V Giroux

**Affiliations:** Universite de Sherbrooke, Sherbrooke, QC, Canada; Universite de Sherbrooke, Sherbrooke, QC, Canada; Universite de Sherbrooke, Sherbrooke, QC, Canada; Universite de Sherbrooke, Sherbrooke, QC, Canada

## Abstract

**Background:**

Esophageal squamous cell carcinoma (ESCC) is deadly with a 5-year survival rate of only 15%. With frequent relapse observed in patients, it is known that exposure to treatment can select for certain types of cells known as cancer stem cells (CSC). Considering that, our laboratory established ESCC cell lines with prolonged exposure to anticancer treatments (radiotherapy, 5-FU chemotherapy and combined therapy). As expected, long-term anticancer treatments result in an increase in CSC proportion. Moreover, metabolic alterations were observed, such as enhanced intracellular lactate concentration. Since 2019, lactate has been linked to a new post-translational modification (PTM) called lactylation. This PTM can affect protein-protein interaction or gene expression regulation but little is known about lactylation in ESCC. In addition to lactate, methylglyoxal (MGO), mainly produced through glycolysis, can also be used as a substrate for this type of modification.

**Aims:**

Investigate the role of lactylation in ESCC.

**Methods:**

Three ESCC cell lines (TE11, TE5 and HCE4), an immortalized normal esophageal cell line (STR) and esophageal organoids derived from a chemically-induced ESCC mouse model were used. Cells were treated with lactate or MGO to increase lactylation. Lactylome was determined by mass spectrometry of peptides pulled down using L-lactyllysine (KLA) beads. Western blot (WB) and immunofluorescence (IF) were also performed with KLA specific antibodies.

**Results:**

ESCC and normal cell lines are more sensitive to MGO than lactate to induce lactylation. Interestingly, when compared to normal samples, tumor organoids and ESCC cell lines show increased Glo1 and LDHA expression, 2 conversion enzymes important to produce lactylation substrates. Lactylome analysis of TE11 cells treated or not with MGO showed an increase in several lactylated proteins such as ACTB and CTNNA1. Treatment with MGO also modulated enrichment in biological processes, shifting from hits related to chromatin assembly and mRNA regulation to metabolic processes such as RNA, peptides and glycolytic metabolic processes. Interestingly, the predicted localization of proteins was more cytosolic in MGO-treated cells vs untreated, which was more nuclear. IF with a KLA antibody confirmed those predictions. Finally, lactylation can occur on multiple lysines of proteins. For example, lactylation could be detected on 21 and 16 lysines for NCL or HISTH1B, respectively. Interestingly, lactylation could be detected up to 5 times on the same peptides, for example on CHD5 and CNGA1, which could severely affect the function of that protein domain.

**Conclusions:**

Our results showed that esophageal cells are sensitive to MGO as a substrate of lactylation and that MGO can induce the lactylation of proteins located in the cytoplasm, hence the difference in localization and associated biological processes.

**Funding Agencies:**

CAGCIHR, TRIANGLE, Chaires de recherche du Canada, CRCHUS, Université de Sherbrooke

